# A global 0.05° dataset for gross primary production of sunlit and shaded vegetation canopies from 1992 to 2020

**DOI:** 10.1038/s41597-022-01309-2

**Published:** 2022-05-16

**Authors:** Wenjun Bi, Wei He, Yanlian Zhou, Weimin Ju, Yibo Liu, Yang Liu, Xiaoyu Zhang, Xiaonan Wei, Nuo Cheng

**Affiliations:** 1 Jiangsu Provincial Key Laboratory of Geographic Information Science and Technology, Key Laboratory for Land Satellite Remote Sensing Applications of Ministry of Natural Resources, School of Geography and Ocean Science, Nanjing University, Nanjing, Jiangsu 210023 China; 2 International Institute for Earth System Science, Nanjing University, Nanjing, 210023 China; 3 State Key Laboratory of Remote Sensing Science Jointly Sponsored by Beijing Normal University and Aerospace Information Research Institute, Chinese Academy of Sciences, Beijing, 100854 China; 4 Jiangsu Center for Collaborative Innovation in Geographical Information Resource Development and Application, Nanjing, Jiangsu 210023 China; 5 Jiangsu Key Laboratory of Agricultural Meteorology, School of Applied Meteorology, Nanjing University of Information Science and Technology, Nanjing, 210044 China; 6 State Key Laboratory of Resources and Environmental Information System, Institute of Geographic Sciences and Natural Resources Research, Chinese Academy of Sciences, Beijing, 100101 China

**Keywords:** Ecological modelling, Climate change, Carbon cycle, Carbon cycle

## Abstract

Distinguishing gross primary production of sunlit and shaded leaves (GPP_sun_ and GPP_shade_) is crucial for improving our understanding of the underlying mechanisms regulating long-term GPP variations. Here we produce a global 0.05°, 8-day dataset for GPP, GPP_shade_ and GPP_sun_ over 1992–2020 using an updated two-leaf light use efficiency model (TL-LUE), which is driven by the GLOBMAP leaf area index, CRUJRA meteorology, and ESA-CCI land cover. Our products estimate the mean annual totals of global GPP, GPP_sun_, and GPP_shade_ over 1992–2020 at 125.0 ± 3.8 (mean ± std) Pg C a^−1^, 50.5 ± 1.2 Pg C a^−1^, and 74.5 ± 2.6 Pg C a^−1^, respectively, in which EBF (evergreen broadleaf forest) and CRO (crops) contribute more than half of the totals. They show clear increasing trends over time, in which the trend of GPP (also GPP_sun_ and GPP_shade_) for CRO is distinctively greatest, and that for DBF (deciduous broadleaf forest) is relatively large and GPP_shade_ overwhelmingly outweighs GPP_sun_. This new dataset advances our in-depth understanding of large-scale carbon cycle processes and dynamics.

## Abstract (summary)

**Table:** 

Measurement(s)	gross primary production of sunlit and shaded vegetation canopies
Technology Type(s)	the revised TL-LUE model
Sample Characteristic - Environment	carbon cycling
Sample Characteristic - Location	terrestrial ecosystem

## Background & Summary

Gross primary production (GPP) is a vital component of the terrestrial carbon budget and plays a prominent role in the global carbon cycle^1–4^. Accurate estimation of terrestrial GPP is critical for understanding the interaction between the terrestrial biosphere and the atmosphere in the context of global climate change^5,6^, projecting future change^7^, and informing climate policy decisions^8^. Therefore, characterizing the spatiotemporal variation of GPP^9^ is a key issue in the climate change study.

GPP is closely related to vegetation types^10–12^, meteorological factors^13–17^, soil moisture^18,19^, and other factors. In particular, GPP is affected by vegetation canopy structures^12,20,21^, e.g., sunlit and shaded leaves. Sunlit leaves can absorb direct and diffuse radiation simultaneously, and light saturation is easy to occur when the radiation is high, while shaded leaves can only absorb diffuse radiation and the absorbed radiation intensity is generally between the light compensation point and the light saturation point^22–24^. The two components of GPP derived from sunlit (GPP_sun_) and shaded leaves (GPP_shade_) have drawn increasing attentions recently due to two reasons. First, commonly used sun-induced chlorophyll fluorescence (SIF), which is strongly correlated with GPP at various scales^25–30^, is mainly emitted from sunlit leaves^31–34^ and GPP_sun_ can also be used to retrieve key photosynthetic parameters such as maximum carboxylation velocity (V_cmax_)^35^. Besides, the contribution of GPP_shade_ to the total GPP increased with the increases of leaf area index (LAI) and diffuse radiation ratio^36,37^. Therefore, it is of great importance to distinguish GPP_shade_ and GPP_sun_ respectively for building an improved SIF-GPP relationship and for obtaining high-precision photosynthetic parameters to feed carbon cycle models. Second, some process-based terrestrial biosphere models simulate GPP_sun_ and GPP_shade_ individually, but there is a lack of credible, large-scale, and long-time series GPP_sun_ and GPP_shade_ products for validating model outputs. Thus, such products can support exploring the similarities and differences of sunlit and shaded leaves contributing to GPP or SIF, to further excavate the interior ecological mechanism of different carbon cycle processes and advance carbon cycle modelling.

Light use efficiency (LUE) models have the advantages of few required parameters, and easy to digest remote sensing data^38,39^. They have been established as a popular method to estimate regional and global carbon fluxes^40–44^. By incorporating satellite-derived land surface variables into LUE models, several global data products (e.g. MOD17A2) have been produced^45–47^. Considering the difference in solar radiation absorption and LUE of leaves within a canopy, the two-leaf LUE (TL-LUE) model divides the vegetation canopy into shaded and sunlit leaves and calculates GPP separately for these two portions^48^. Therefore, the TL-LUE model significantly reduces the sensitivity to sky conditions, effectively alleviates the systematic underestimation of GPP under low solar radiation by the MOD17 model, and improves the simulation accuracy of GPP^48,49^.

In the previous version of the TL-LUE model, the CO_2_ fertilization effect (CFE), the enhancement of vegetation productivity by the increase of CO_2_ concentration, is not included. It’s well-known that global atmospheric CO_2_ concentration has continued to rise, increasing about 17% during 1992–2020, and the increase of atmospheric CO_2_ substantially enhance global GPP^16^. CFE on the global terrestrial carbon exchange has attracted widespread attentions^4,16,50^, but has rarely been considered in LUE models. Thus, it is imperative to include the change of atmospheric CO_2_ concentration in GPP estimation with LUE models. Based on the previous version of TL-LUE model, this study additionally includes atmospheric CO_2_ concentration regulation scalar (C_S_) and replaces temperature regulation scalar (T_S_) with that used in the Terrestrial Ecosystem Model (TEM) model^51^ to capture negative effects of low and high temperatures on GPP. Eddy covariance carbon flux measurements of 68 sites (480 site years) and 25 sites (170 site years) from the FLUXNET2015 dataset were used to calibrate and validate the revised TL-LUE model, respectively.

**Fig. 1:**
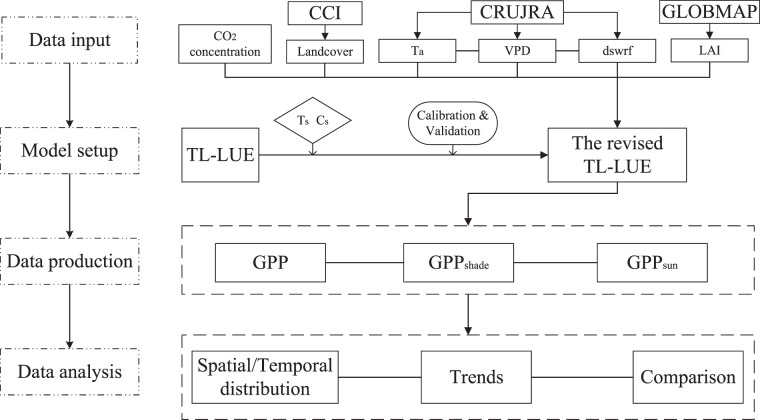
Workflow of the study. CCI represents European Space Agency Climate Change Initiative Land Cover. CRUJRA represents the Climatic Research Unit and Japanese reanalysis. VPD is vapor pressure deficit. T_a_ is air temperature. T_s_ and C_s_ are regulation scalars for temperature and CO_2_ concentration. The dswrf is downward solar radiation flux. TL-LUE is two-leaf light use efficiency model. GPP_sun_ and GPP_shade_ are GPP derived by sunlit and shaded leaves.

This study employs various remote sensing data as model inputs, including European Space Agency Climate Change Initiative Land Cover (ESA-CCI land cover) from 1992 to 2020 and GLOBMAP leaf area index (GLOBMAP-LAI), in conjunction with meteorological data provided by the Climatic Research Unit and Japanese reanalysis (CRUJRA v2.2) dataset. The GPPs are calculated by the revised TL-LUE model (Fig. 1). The temporal resolutions of the dataset are 8-day, monthly and annual, and the spatial resolution is 0.05°×0.05°. During 1992–2020, the estimated mean annual totals of global GPP, GPP_sun_, and GPP_shade_ are 125.0 Pg C a^−1^, 50.5 Pg C a^−1^, and 74.5 Pg C a^−1^, respectively.

## Methods

### Model description

The model used in this study is a revised version of the two-leaf light use efficiency (TL-LUE) model^48^. The revised TL-LUE model adds the atmospheric CO_2_ concentration regulation scalar and modifies air temperature regulation scalar. GPP is divided into GPP_shade_ and GPP_sun_^52^. It is described as Eqs. (1–3):

1 $${\rm{GPP}}={{\rm{GPP}}}_{{\rm{shade}}}+{{\rm{GPP}}}_{{\rm{sun}}}$$

2 $${{\rm{GPP}}}_{{\rm{shade}}}=({{\rm{\varepsilon }}}_{{\rm{msh}}}\times {{\rm{APAR}}}_{{\rm{sh}}})\times {{\rm{T}}}_{{\rm{s}}}\times {{\rm{W}}}_{{\rm{s}}}\times {{\rm{C}}}_{{\rm{s}}}$$

3 $${{\rm{GPP}}}_{{\rm{sun}}}=({{\rm{\varepsilon }}}_{{\rm{msu}}}\times {{\rm{APAR}}}_{{\rm{su}}})\times {{\rm{T}}}_{{\rm{s}}}\times {{\rm{W}}}_{{\rm{s}}}\times {{\rm{C}}}_{{\rm{s}}}$$

where ε_msu_ and ε_msh_ are the maximum light use efficiency of sunlit and shaded leaves (detailed in Table 1), respectively. APAR_su_ and APAR_sh_ are the absorbed PAR of sunlit and shaded leaves, respectively. They were expressed as:

4 $${{\rm{APAR}}}_{{\rm{sh}}}=(1-{\rm{\alpha }})\times \left(\frac{{{\rm{PAR}}}_{{\rm{dif}}}-{{\rm{PAR}}}_{{\rm{dif}},{\rm{u}}}}{{\rm{LAI}}}+{\rm{C}}\right)\times {{\rm{LAI}}}_{{\rm{sh}}}$$

5 $${{\rm{APAR}}}_{{\rm{su}}}=(1-{\rm{\alpha }})\times \left(\frac{{{\rm{PAR}}}_{{\rm{dir}}}\times \cos \,{\rm{\beta }}}{\cos \,\theta }+\frac{{{\rm{PAR}}}_{{\rm{dif}}}-{{\rm{PAR}}}_{{\rm{dif}},{\rm{u}}}}{{\rm{LAI}}}+{\rm{C}}\right)\times {{\rm{LAI}}}_{{\rm{su}}}$$

6 $${{\rm{LAI}}}_{{\rm{su}}}=2\,\cos \,\theta \times \left(1-{{\rm{e}}}^{-\frac{{\rm{LAI}}\times \Omega }{2\cos \theta }}\right)$$

7 $${{\rm{LAI}}}_{{\rm{sh}}}={\rm{LAI}}-{{\rm{LAI}}}_{{\rm{su}}}$$

8 $${\rm{C}}=0.07\times \Omega \times {{\rm{PAR}}}_{{\rm{dir}}}\times (1.1-0.1{\rm{LAI}}){{\rm{e}}}^{-\cos {\rm{\theta }}}$$

9 $${{\rm{PAR}}}_{{\rm{dif}}}={\rm{PAR}}\times (0.7527+3.8453{\rm{R}}-16.316{{\rm{R}}}^{2}+18.962{{\rm{R}}}^{3}-7.0802{{\rm{R}}}^{4})$$

10 $${{\rm{PAR}}}_{{\rm{dir}}}={\rm{PAR}}-{{\rm{PAR}}}_{{\rm{dif}}}$$

11 $${{\rm{PAR}}}_{{\rm{dif}},{\rm{u}}}={{\rm{PAR}}}_{{\rm{dif}}}\times {{\rm{e}}}^{-\frac{0.5\Omega {\rm{LAI}}}{\cos \bar{{\rm{\theta }}}}}$$

12 $$\cos \,\bar{{\rm{\theta }}}=0.537+0.025\times {\rm{LAI}}$$

where α is albedo; θ is the solar zenith angle; β is the leaf angle, which is set as 60°; Ω is clumping index (detailed in Table 1); C is multiple scattered radiation (unit: W m^−2^); PAR_dir_ and PAR_dif_ (unit: W m^−2^) are the incoming direct and diffuse photosynthetically active radiation above the canopy; PAR_dif,u_ (unit: W m^−2^) denotes diffuse PAR under the canopy^48,53^; LAI_su_ and LAI_sh_ are the LAI of sunlit and shaded leaves; R represents the sky clearness index and equals to S/(S_0_ cosθ), where S is solar radiation in W m^−2^, and S_0_ is the solar constant (1367 W m^−2^); $\bar{{\rm{\theta }}}$ is the representative zenith angle for diffuse radiation transmission^53^.

**Table 1:** Parameters used in the revised TL-LUE model.

Vegetation Type	DBF	EBF	ENF	MF	CRO	GRA	OSH	SAV	WET	WSA
ε_msh_(gC MJ^−1^)	3.75 ± 0.52	3.26 ± 0.93	3.40 ± 1.19	3.00 ± 0.66	4.80 ± 1.94	4.57 ± 1.67	3.10 ± 0.42	4.65 ± 0.64	2.53 ± 1.02	2.70
ε_msu_(gC MJ^−1^)	0.92 ± 0.29	1.44 ± 0.64	0.89 ± 0.49	0.80 ± 0.41	1.43 ± 0.75	1.16 ± 0.45	0.65 ± 0.07	3.45 ± 0.64	1.23 ± 0.92	2.60
VPD_max_(kPa)	4.1	4.1	4.1	4.1	4.1	4.1	4.1	4.1	4.1	4.1
VPD_min_(kPa)	0.93	0.93	0.93	0.93	0.93	0.93	0.93	0.93	0.93	0.93
T_opt_(°C)	23.1	25.8	19.7	24.5	23.5	20.9	22.3	25.8	24.2	26.2
albedo(α)^49,95,96^	0.18	0.18	0.15	0.17	0.23	0.23	0.16	0.18	0.23	0.23
Clumping index(Ω)^97^	0.8	0.8	0.6	0.7	0.9	0.9	0.8	0.8	0.9	0.8

DBF: deciduous broadleaf forest; EBF: evergreen broadleaf forest; ENF: evergreen needleleaf forest; MF: mixed forest; CRO: cropland; GRA: grasslands; OSH: open shrublands; SAV: savannas; WET: wetlands; WSA: woody savannas.

The regulation scalars of temperature (T_s_)^52^, water stress (W_s_)^48^, and atmospheric CO_2_ concentration (C_s_)^54^ are calculated as follows:

13 $${{\rm{T}}}_{{\rm{s}}}=\frac{({\rm{T}}-{{\rm{T}}}_{{\rm{\max }}})\times ({\rm{T}}-{{\rm{T}}}_{{\rm{\min }}})}{({\rm{T}}-{{\rm{T}}}_{{\rm{\max }}})\times ({\rm{T}}-{{\rm{T}}}_{{\rm{\min }}})-{({\rm{T}}-{{\rm{T}}}_{{\rm{opt}}})}^{2}}$$

14 $${{\rm{W}}}_{{\rm{s}}}=\frac{{{\rm{VPD}}}_{{\rm{\max }}}-{\rm{VPD}}}{{{\rm{VPD}}}_{{\rm{\max }}}-{{\rm{VPD}}}_{{\rm{\min }}}}$$

15 $${{\rm{C}}}_{{\rm{s}}}=\frac{{{\rm{C}}}_{{\rm{i}}}-{\Gamma }^{* }}{{{\rm{C}}}_{{\rm{i}}}+2{\Gamma }^{* }}$$

where the maximum (T_max_) and minimum temperatures for vegetation photosynthesis (T_min_) were set to 313.15 K and 273.15 K, respectively^49^. The optimum temperature for vegetation photosynthesis (T_opt_) is set as the average of different types in Huang *et al*.^55^ (details in Table 1). VPD_max_ and VPD_min_ are the VPD when GPP reaches the maximum and minimum, respectively^38^. If the value of VPD is greater than or equal to VPD_max_, W_s_ is equal to 0 and if the value of VPD is less than or equal to VPD_min_, W_s_ is set to 1^48^. Γ^*^ is the CO_2_ compensation point in the absence of dark respiration (calculated by Eq. 16)^54^; C_i_ is the intercellular concentration of CO_2_ (ppm).

16 $${\Gamma }^{* }=4.22{\times {\rm{e}}}^{\frac{37830({\rm{T}}-298.15)}{298.15{\rm{RT}}}}$$

17 $${{\rm{C}}}_{{\rm{i}}}={{\rm{C}}}_{{\rm{a}}}\times {\rm{\chi }}$$

18 $${\rm{\chi }}=\frac{{\rm{\xi }}}{{\rm{\xi }}+\sqrt{{\rm{VPD}}}}$$

19 $${\rm{\xi }}=\sqrt{\frac{356.51{\rm{K}}}{1.6{{\rm{\eta }}}^{\ast }}}$$

20 $${\rm{K}}={{\rm{K}}}_{{\rm{c}}}\times \left(1+\frac{{{\rm{P}}}_{{\rm{o}}}}{{{\rm{K}}}_{{\rm{o}}}}\right)$$

21 $${{\rm{K}}}_{{\rm{c}}}=39.97\times {{\rm{e}}}^{\frac{79.43\times ({\rm{T}}-298.15)}{298.15{\rm{RT}}}}$$

22 $${{\rm{K}}}_{{\rm{o}}}=27480\times {{\rm{e}}}^{\frac{36.38\times ({\rm{T}}-298.15)}{298.15{\rm{RT}}}}$$

where C_a_ is the atmospheric CO_2_ concentration (using NOAA global monthly mean CO_2_ concentration at the unit of ppm), and χ is the ratio of leaf-internal to ambient CO_2_^56^. K is the Michaelis-Menten coefficient of Rubisco and η* is the viscosity of water relative to its value at 25 °C (0.8903)^57^. K_c_ and K_o_ are the Michaelis-Menten coefficients of Rubisco for CO_2_ and O_2_, respectively, and P_o_ is the partial pressure of O_2_ (21 kPa)^56^. R is the molar gas constant (8.314 J mol^−1^ K^−1^).

### Input data and processing

Eddy covariance flux measurements from the FULLSET daily product in the FLUXNET2015 dataset (https://fluxnet.org) were used for model calibration and validation. We selected site years data according to the following requirements: the missing observations of air temperature (TA_F), vapor pressure deficit (VPD_F), CO_2_ mole fraction (CO2_F_MDS), incoming photosynthetic photon flux density (PPFD_IN), or shortwave radiation (SW_IN_F), gross primary production (GPP_DT_CUT_MEAN) in one year are less than 2 months. A linear interpolation was applied to fill the individual missing values, which accounted for about 2% of the total measurements. About 75% of the sites were randomly selected to calibrate the revised TL-LUE model parameters for each vegetation type, and the remaining sites were applied for parameters validation. The sites and years selected for calibration and validation are detailed in Table S1. The shortwave-to-PAR conversion parameter in global was estimated to vary between 0.39 to 0.53^58–60^. With the comparison between measurements of PAR and shortwave radiation in sites, 0.43 is most suitable for this study. The spatial distribution of calibration and validation sites is shown in Figure S1.

The land cover dataset we used is European Space Agency Climate Change Initiative Land Cover (ESA-CCI land cover) at a 300 m spatial resolution for every year from 1992 to 2020 (https://cds.climate.copernicus.eu/cdsapp#!/dataset/satellite-land-cover). We resampled the raw data to 0.05°×0.05° using nearest neighbour resampling. ESA-CCI land cover dataset uses the United Nations Land Cover Classification System (LCCS), thus, we converted them to match the International Geosphere-Biosphere Program land cover scheme (IGBP)^61^. In particular, ESA-CCI land cover provides land cover data for the years before 2000, which makes it possible to study changes in GPP caused by changes in land cover types over long-term series.

GLOBMAP leaf area index (GLOBMAP-LAI)^62^ as a model input is available at 0.0727° spatial resolution for every 8 days (2001–2020) and half-month (1992–2000) from 1992 to 2020. GLOBMAP LAI (Version 3) provides a consistent long-time LAI product (1981–2020, continuously updated) by quantitative fusion of Moderate Resolution Imaging Spectroradiometer (MODIS) and historical Advanced Very High Resolution Radiometer (AVHRR) data. The long-term LAI series was made up by combination of AVHRR LAI (1981–2000) and MODIS LAI (2001–2020). MODIS LAI series was generated from MODIS land surface reflectance data (MOD09A1)^63^ based on the GLOBCARBON LAI algorithm^64^. The relationships between GIMMS NDVI and MODIS LAI were established pixel by pixel using the two data series during overlapped period (2000–2006). And the AVHRR LAI^65^ back to 1981 was estimated from historical AVHRR observations based on these pixel-level relationships. The clumping effects was considered at the pixel level by employing global clumping index map at 500 m resolution^66^. The cloud mask for the MOD09A1 data were created by a new cloud detection algorithm based on time series surface reflectance observations^67^. GLOBMAP-LAI has been smoothed by locally adjusted cubic spline capping approach^68^. We resampled them to 0.05°×0.05° for the model. Additionally, we extracted the LAI of each site from GLOBMAP-LAI (500 m, 8-day) for model calibration and validation.

The Climatic Research Unit and Japanese reanalysis (CRUJRA) version 2.2 data^69^ provided 6-hourly at 0.5° resolution meteorological variables, such as downward solar radiation flux (dswrf, unit: J m^−2^ 6 h^−1^), specific humidity(spfh, unit: kg kg^−1^), the temperature at 2 m (tmp, unit: K), pressure (pres, unit: Pa), from 1992 to 2020. Daily dswrf used in this study (unit: J m^−2^ d^−1^) was converted from the CRUJRA dswrf dataset by summing four 6-hourly data per day. Temperature (unit: K) and vapor pressure deficit (VPD, unit: kPa) by taking the mean of the 6-hourly data of each day in CRUJRA. The daily dswrf was corrected with site shortwave radiation data. The global monthly CO_2_ concentration (unit: ppm) is available on www.esrl.noaa.gov/gmd/ccgg/trends/. VPD was calculated using specific humidity, pressure, and temperature as Eqs. (23, 24):

23 $${\rm{VPD}}={{\rm{VP}}}_{{\rm{sat}}}-{\rm{spfh}}\times \frac{{\rm{pres}}/1000}{0.622+{\rm{spfh}}\times 0.378}$$

24 $${{\rm{VP}}}_{{\rm{sat}}}=0.61121\times {{\rm{e}}}^{\left(18.678-\frac{{{\rm{t}}}_{{\rm{air}}}}{234.5}\right)\times \frac{{{\rm{t}}}_{{\rm{air}}}}{257.14+{{\rm{t}}}_{{\rm{air}}}}}$$

where t_air_ is the air temperature at the unit of °C. VP_sat_ means the saturated vapor pressure (kPa); spfh represents specific humidity(kg kg^−1^); press is pressure(Pa).

### Calibration of model parameters

The maximum LUEs of the sunlit (ε_msu_) and shaded (ε_msh_) leaves spatially differ due to the changes in vegetation canopy structures and vegetation species types^70^, which leads to the distinct ε_msh_ and ε_msu_ of different vegetation types. To reduce the GPP simulation bias caused by ε_msh_ and ε_msu_ of sunlit and shaded leaves, the daily data of 68 sites (480 site years) in the FLUXNET2015 dataset (details in Table S1) were used for parameter optimization with the shuffled complex Evolution-University of Arizona method^49,71^. The agreement index (d) was used as the optimization criterion. This index was widely used in parameter optimization^49,72^. It ranges from 0 (complete disagreement) to 1 (complete agreement). Parameter values identified when d maximizes are optimization results. The calculation of d is as Eq. (25):

25 $${\rm{d}}=1-{\sum }_{{\rm{n}}=1}^{{\rm{m}}}{\left({{\rm{E}}}_{{\rm{n}}}-{{\rm{M}}}_{{\rm{n}}}\right)}^{2}/{\sum }_{{\rm{n}}=1}^{{\rm{m}}}{\left(| {{\rm{E}}}_{{\rm{n}}}-\bar{{\rm{M}}}| +| {{\rm{M}}}_{{\rm{n}}}-\bar{{\rm{M}}}| \right)}^{2}$$

where m is the number of all measurements; E_n_ and M_n_ are the n^th^ estimation and measured GPP, respectively. $\bar{{\rm{M}}}$ represents the mean of all measured GPP values.

The average ε_msh_ and ε_msu_ values with standard deviation of all vegetation types are shown in Table 1. The parameters of deciduous needleleaf forests (DNF) were consistent with DBF settings in modelling. The R^2^ of observed GPP (GPP_EC_) and estimated GPP (GPP) in WSA and SAV were 0.39 and 0.41, respectively, and the R^2^ values of other vegetation types were all above 0.5, in which the R^2^ of DBF was the highest (0.89). The calibration results of different vegetation types are shown in Figure S2.

## Data Records

The dataset provides global gridded 0.05° GPP, GPP_shade_ and GPP_sun_ at three temporal resolutions (8-day, monthly, annual) from 1992 to 2020. The units are g C m^−2^ (8day)^−1^, g C m^−2^ month^−1^ and g C m^−2^ a^−1^, respectively. We divided the dataset into 29 folders by year, where each folder contains the data for the year at three temporal scales. The files were named as “GGGG_v21_TTTT.tif” and stored in the GeoTiff format, where GGGG represents GPP, GPP_shade_ and GPP_sun_. For the 8-day scale, TTTT represents year and DOY (e.g. Shade_GPP_v21_1999_249.tif). For the one-month scale, TTTT represents year and month (e.g. Sun_GPP_v21_1999_01.tif). For the annual scale, TTTT represents only year (e.g. GPP_v21_1999.tif). The scale factor of the monthly data is 0.1, that of the 8-day data is 0.01. The data type of the monthly and 8-day data is 16-bit integer, and that of the annual data is double. All datasets are publicly available from the DRYAD repository 10.5061/dryad.dfn2z352k^73^.

**Fig. 2:**
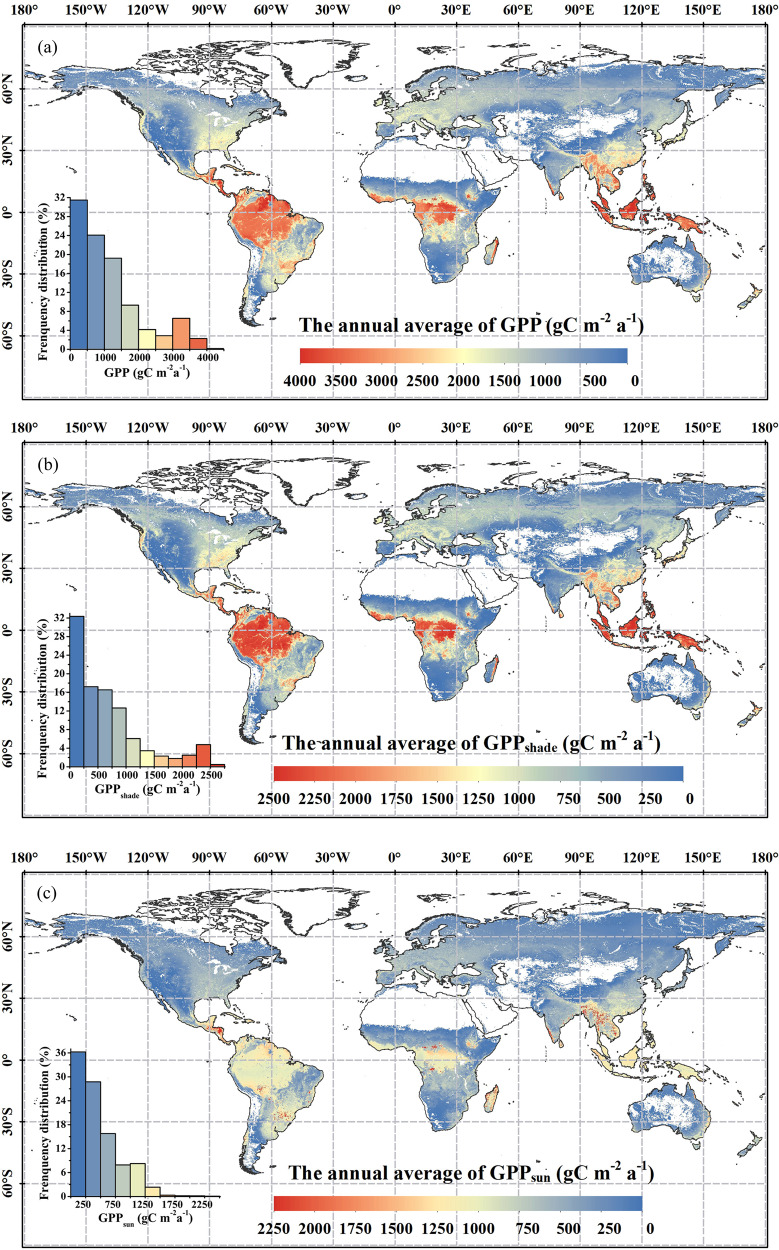
Spatial distribution of the annual average (**a**) GPP, (**b**) GPP_shade_, and (**c**) GPP_sun_ from 1992 to 2020.

The high annual average GPP values were mainly distributed in the Amazon, Indonesia and Congo Basin in the low latitude regions, which was about 3500 g C m^−2^ a^−1^. The relatively high GPP (~2000 g C m^−2^ a^−1^) occurred in Southeast Asia, the southeast of North and South America, and south central Africa, and the GPP that ranged from 0 to 1500 g C m^−2^ a^−1^ accounted for 74.7% of global vegetation cover. The shaded and sunlit GPP (GPP_shade_ and GPP_sun_) had similar spatial distribution with GPP, but the value of GPP_sun_ was lower than GPP_shade_ near the equator (Fig. 2b,c). GPP_shade_ that ranged from 0 to 1000 g C m^−2^ a^−1^ accounted for 78.7% of global vegetation cover, and GPP_sun_ that ranged from 0 to 750 g C m^−2^ a^−1^ accounted for 80.8% of global vegetation cover.

**Fig. 3:**
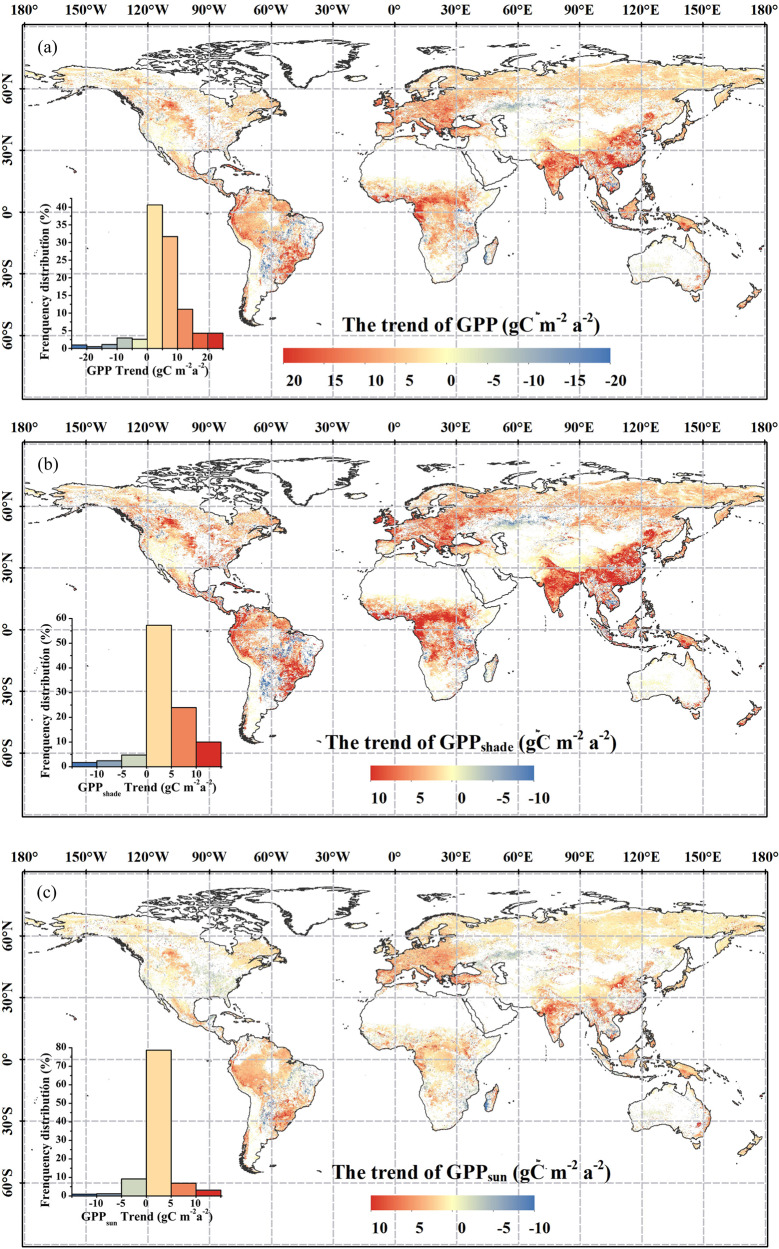
Spatial distribution of the trend of (**a**) GPP, (**b**) GPP_shade_, and (**c**) GPP_sun_ during 1992 to 2020. The results have removed the value which is not significant (p > 0.05).

From 1992 to 2020, the GPP of Malaysia, Southeast Asia, Indian Peninsula, central Africa, north and southeast South America, and central Europe all showed an obvious increasing trend, and the rate was close to 20 g C m^−2^ a^−2^. The GPP scattered in central South America, east Africa, Central Asia, and Southeast Asia showed a significant decreasing trend, and the change rate was near to −20 g C m^−2^ a^−2^ (Fig. 3a). For GPP_shade_, eastern and southern Asia, central Europe, central and western Africa, northwest and southeast South America showed a significant increasing trend, and the change rate was above 10 g C m^−2^ a^−2^, which was consistent with the trend of GPP. Besides, areas with reduced GPP_shade_ (trend < −10 g C m^−2^ a^−2^) were sporadically distributed in Central Asia, Central South America, Eastern Africa, and Southeast Asia (Fig. 3b). For GPP_sun_, central and southeastern South America, southeastern Asia, and Europe showed the most obvious increase, with a rate of change of around 10 g C m^−2^ a^−2^, which was similar to the spatial distribution of GPP and GPP_shade_ trends. However, the value of GPP_sun_ was lower than GPP_shade_ (Fig. 3b,c). The vast majority of global vegetation cover with significant change exhibits an increasing trend (GPP trend >0: 92.0%, GPP_shade_ trend >0: 91.2%, and GPP_sun_ trend >0: 88.7%). In general, most global GPP, GPP_shade_, and GPP_sun_ revealed increasing trends (Fig. 3).

**Fig. 4:**
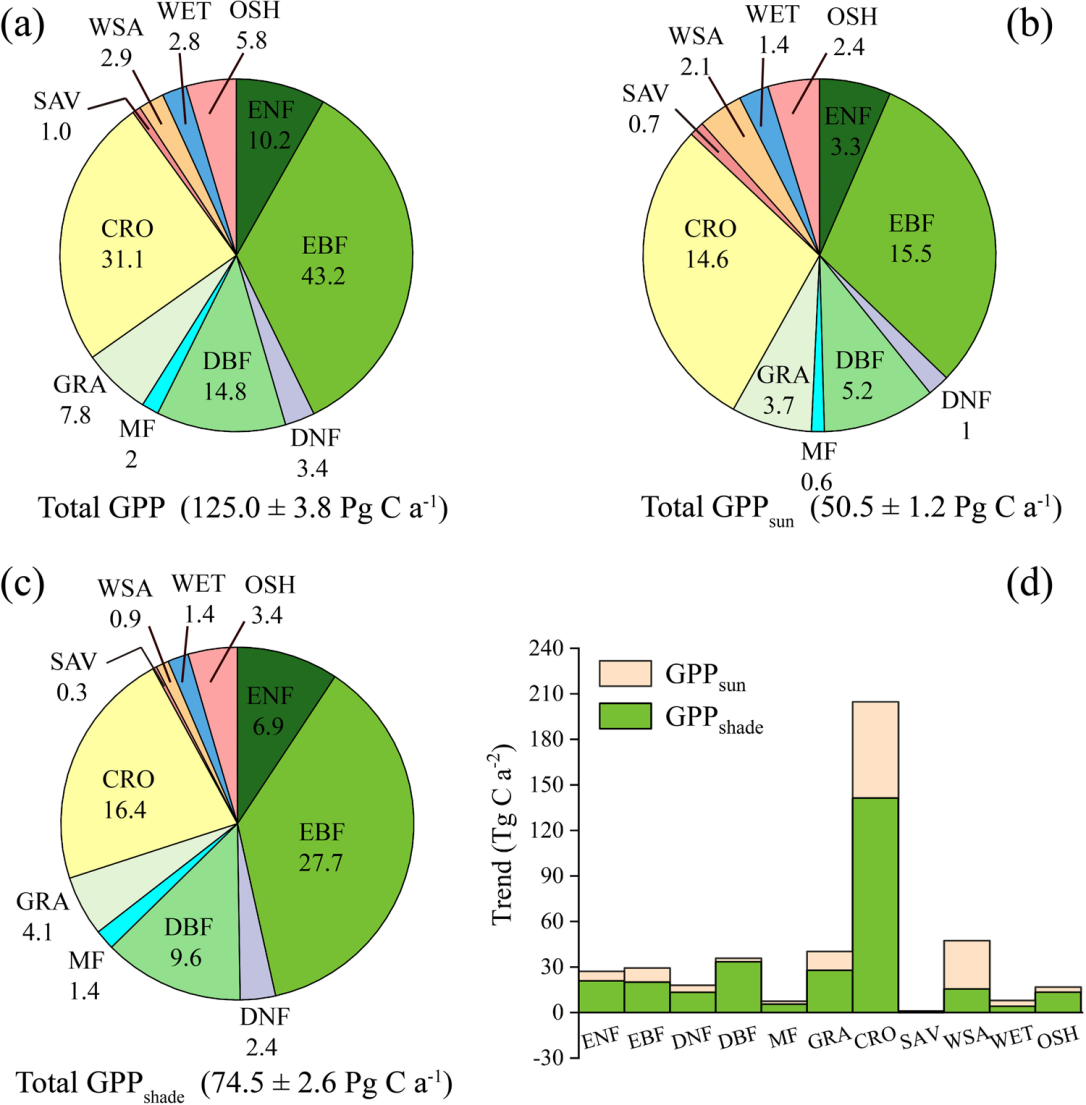
The mean annual totals of (**a**) GPP, (**b**) GPP_sun_, (**c**) GPP_shade_ and (**d**) the trend of GPP_shade_ and GPP_sun_ from 1992 to 2020 for different vegetation types over the globe.

The mean annual totals of global GPP, GPP_sun_, and GPP_shade_ from 1992 to 2020 are 125.0 Pg C a^−1^, 50.5 Pg C a^−1^ and 74.5 Pg C a^−1^, respectively. Overall, the GPP proportions of individual vegetation types to total were similar for global GPP, GPP_sun_, and GPP_shade_. Among the 11 vegetation types, EBF contributed most GPP, followed by CRO. These two types accounted for more than half of the total GPP, GPP_sun_, and GPP_shade_ (Fig. 4). The GPP of SAV, MF, WET, and WSA were 1.0 Pg C a^−1^, 2.0 Pg C a^−1^, 2.8 Pg C a^−1^, and 2.9 Pg C a^−1^, respectively, which were relatively low (Fig. 4a). The GPP_sun_ were close to GPP_shade_ for CRO, GRA, and WET, while the GPP_sun_ were higher than GPP_shade_ for SAV and WSA. The GPP_shade_ of ENF, EBF, DNF, DBF, MF, and OSH with relatively complicated canopy structures were much higher than their GPP_sun_ (Fig. 4b,c). Overall, GPP_shade_ played a key role in GPP for forest types, while GPP_sun_ was greater than GPP_shade_ for non-forest types. In total, GPP_shade_ contributes more to GPP than GPP_sun_. In addition, the GPP of all vegetation types showed an increasing trend. With one exception that the GPP_sun_ of SAV showed a decreasing trend, the GPP_sun_ and GPP_shade_ of other vegetation types showed an increasing trend, among which GPP (also GPP_sun_, and GPP_shade_) of CRO showed the distinctively greatest increasing trend. Except WSA, the increasing trend of GPP_shade_ of other vegetation types is greater than that of GPP_sun_ (Fig. 4d). It’s worth noting that for DBF, the increasing trend of GPP is relatively large, and GPP_shade_ overwhelmingly outweighs GPP_sun_. MF shows a similar phenomenon, but the overall trend is smaller.

## Technical Validation

### Validation of model parameters

**Fig. 5:**
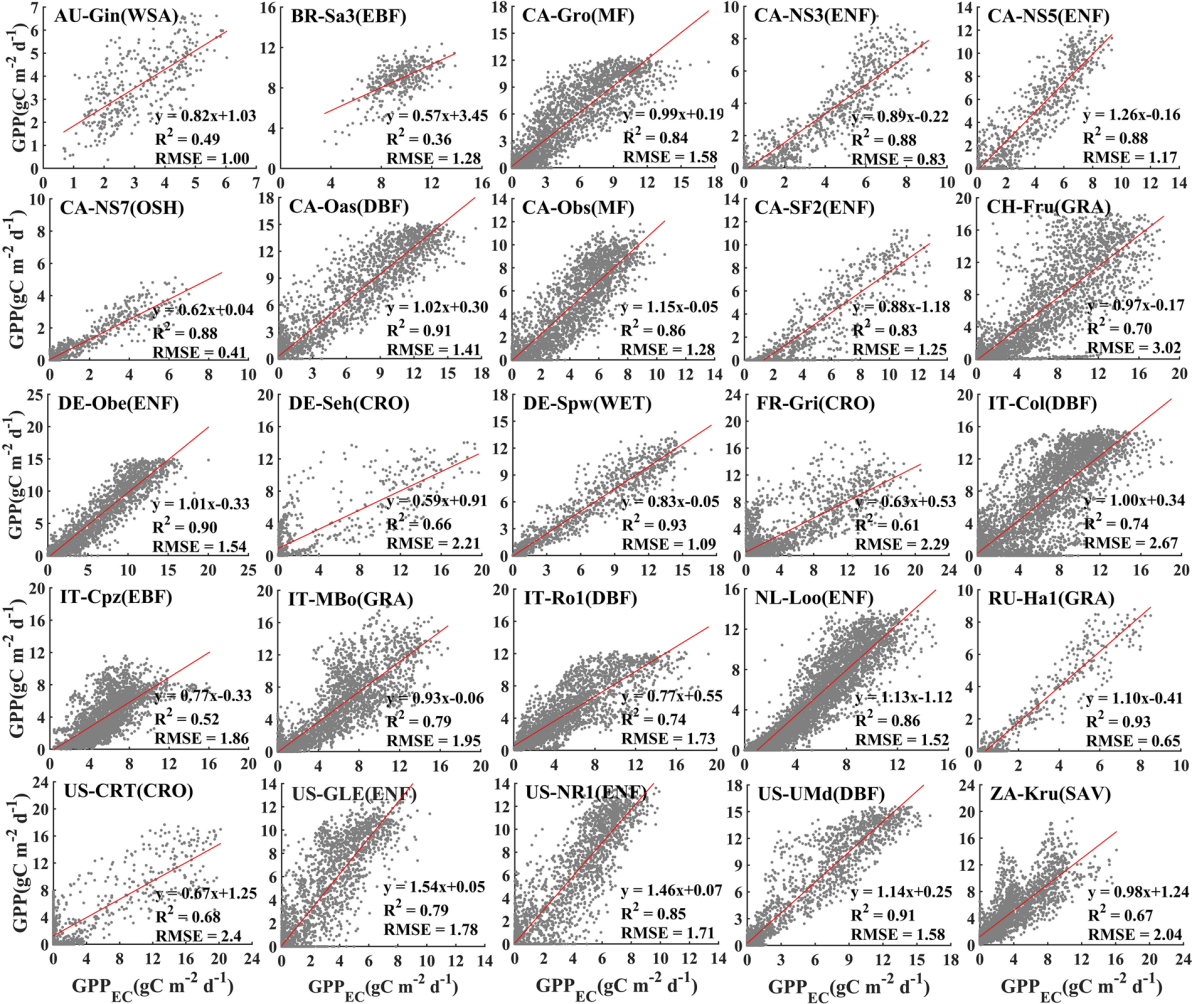
Validation of daily GPP estimated by the revised TL-LUE (GPP) model with tower measurements (GPP_EC_) at 25 FLUXNET sites. The revised TL-LUE model was driven by average optimized parameters for different vegetation types, tower-based meteorological data, and smoothed LAI.

Carbon flux data of 25 sites (170 site years) from the FLUXNET2015 dataset were selected (detailed in Table S1) for model validation. The comparison between GPP that estimated by the revised TL-LUE model with optimized ε_msu_ and ε_msh_, and GPP measurements (GPP_EC_) at each flux site is shown in Fig. 5. The revised TL-LUE model performed well in estimation of GPP for all vegetation types. All sites have R^2^ above 0.5, except for AU-Gin (WSA) (R^2^ = 0.49) and BR-Sa3 (EBF) (R^2^ = 0.36).

### Comparisons with other global GPP products

**Fig. 6:**
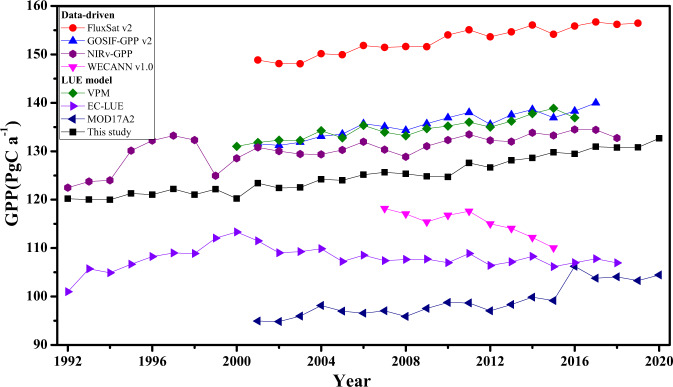
Comparison of global annual GPP totals estimated from a set of global remote sensing GPP products.

Previous studies have shown that the differences in GPP estimation are usually caused by different model structures, parameter settings, and input data^74–76^. Here, we compare our global annual GPP with several global GPP products derived from remote sensing models, including data-driven models and LUE models (Fig. 6).

For data-driven GPP products, Li *et al*.^77^ used SIF-GPP relationship and SIF observed by the Orbiting Carbon Observatory-2 (OCO-2) to obtain global GPP, which named GOSIF GPP (135.5 ± 8.8 Pg C a^−1^, 2001–2017). And the WECANN product was produced using an artificial neural network (ANN) with SIF and other data sources as inputs^78^. The WECANN-GPP showed an obvious decreasing trend between 2007 and 2015, with a range of 110.1 to 118.2 Pg C a^−1^. FluxSat GPP^79^, which was generated using satellite data-driven models based on the LUE framework, ranged from 148.1 to 156.7 Pg C a^−1^ during 2001 to 2019. In addition, a few recent studies have identified that there is a strong spatio-temporal correlation between near-infrared reflectance (NIRv) and GPP, suggesting that NIRv can be employed to estimate the GPP of vegetation^13,79^. The global total of GPP estimated from NIRv by Wang *et al*.^80^ was 130.5 ± 3.2 Pg C a^−1^ during 1992–2018, close to our estimate. For the LUE models, the range of GPP obtained by the improved EC-LUE model^81^ was 106.4~118.3 Pg C a^−1^ (1992–2018), and that by VPM model was 131.2~140.0 Pg C a^−1^ (2000–2016). The range of MOD17A2H.006^82^ was 94.8~106.2 Pg C a^−1^ (2001–2020). The global annual GPP of FluxSat was the highest, and that of MOD17A2 was the lowest. Our estimated global GPP ranged from 120.02 to 132.65 Pg C a^−1^, placing at the middle of the various GPP products. Anav *et al*.^76^ suggested that previous observation-constrained estimates of global GPP^45,83^ (e.g. based on either δ^18^O measurements of atmospheric CO_2_ or eddy covariance flux upscaling) was at 120 Pg C a^−1^ for the period before 2010. Thus, our estimate agreed reasonably well with such estimates.

In addition, our estimated global GPP showed an overall increasing trend, which is consistent with most other GPP products. Only WECANN-GPP and EC-LUE GPP (after 2000) showed a significant declining trend. The declining trend of WECANN obviously associated with the degradation of GOME-2 SIF sensor, and the GOME-2 SIF data used for training was not corrected^84^.

### Uncertainties

Previous studies have indicated that different LAI products lead to clear differences in estimated GPP^85^, and the uncertainties of various LAI products are higher in low-latitude areas^86,87^. Since the land cover used by GLOBMAP-LAI is different from that used in this study, the corresponding LAI values of a small amount pixels (<0.01%) for SAV and WSA in low latitude areas are relatively high, which lead to abnormally high estimated GPP. These anomalies are not processed because of remaining the authenticity of data.

The model parameters varied in different areas, due to the plant species included in the same vegetation type. In particular, the LUE of C3 and C4 crops was greatly discrepant as many previous studies proved^88–91^. According to the restriction of ESA-CCI land cover data, C3 and C4 plants could not be distinguished, so the GPP of C3 and C4 crops cannot be gained separately. The LAI of each site extracted from GLOBMAP-LAI with a resolution of 500 m cannot completely match the flux tower data scale, which cause uncertainty in the parameter optimization. Simultaneously, the eddy covariance measurements also have some uncertainties, which inevitably affected the parameter optimization.

### Potential benefits and usages of this dataset

#### Facilitate researches on the relationship between SIF and GPP

As is known, SIF signals come mainly from sunlit leaves^31,92^. Most of the current studies on the relationship between SIF and the photosynthesis of sunlit leaves are at canopy and leaf scales, while similar studies at large scales are currently rare due to the lack of publicly available global or regional GPP datasets for sunlit leaves. In addition, it is known that there is a link between SIF and GPP across biome types^34^, but the relationship is not well quantified. The GPP_sun_ and GPP provided in this study may help to explore the relationship between SIF and GPP_sun_ or GPP in different ecosystem types at large scales.

#### Facilitate researches on the interactions between solar radiation and terrestrial carbon cycling

Compared with direct radiation, the increase of diffuse radiation can effectively promote carbon fixation^93^. Shaded leaves make more effective use of diffuse radiation^48^, and GPP_shade_ plays a major role in the vegetation areas with more sheltered leaves or cloudy conditions^94^. The GPP_shade_ and GPP_sun_ cannot be directly measured over large regions. The GPP_shade_ and GPP_sun_ estimated by the revised TL-LUE model can capture the contribution of sunlit and shaded leaves to GPP in long-term and at large scales, and make it possible to quantify the carbon fixation increase (or decrease) influenced by the change in radiation fraction over a long period and at large scales, which facilitates further investigations on the interactions between radiation and terrestrial carbon cycling.

#### Facilitate researches on the dynamics, processes and drivers of GPP at large scales

This study provides 8-day and monthly GPP from 1992 to 2020, which allows for studying the changes in seasonal cycles (e.g. amplitude and phase changes of growing season) of GPP and its processes (GPP_sun_ and GPP_shade_) over many years. In addition, we employed a long-term ESA-CCI land cover data since 1992 (while most other dataset uses MODIS land cover since 2000) with the consideration of year to year land cover changes, enabling it to characterize the impact of land cover change on GPP. The dataset would help to dig the underlying mechanisms of climate and human impacts on global terrestrial GPP.

## Usage Notes

In the dataset, in order to ensure the authenticity, we did not delete or modify a small number of abnormally high values. Therefore, when using this dataset, you can set thresholds to remove the anomalies.

## Supplementary information

supplementary

## Data Availability

We used the MATLAB 2020b for data processing. The core codes for the study are available at https://github.com/BiWenjunnju/code_TL.git.
