# Supplementary material for: A global 0.05° dataset for gross primary production of sunlit and shaded vegetation canopies from 1992 to 2020
Source: Sci Data. 2022 May 16;9:213. doi: 10.1038/s41597-022-01309-2 (PMC9110750; doi:10.1038/s41597-022-01309-2)
Supplement: Supplementary file 1 — supplementary [file 41597_2022_1309_MOESM1_ESM.docx]

Supporting Information for

**A global 0.05° dataset for gross primary production of sunlit and shaded vegetation canopies from 1992 to 2020**

Wenjun Bi^1^, Wei He^2,3^, Yanlian Zhou^1,4^*, Weimin Ju^2,4^, Yibo Liu^5^, Yang Liu^6^, Xiaoyu Zhang^1^, Xiaonan Wei^1^, Nuo Cheng^1^

1. Jiangsu Provincial Key Laboratory of Geographic Information Science and Technology, Key Laboratory for Land Satellite Remote Sensing Applications of Ministry of Natural Resources, School of Geography and Ocean Science, Nanjing University, Nanjing, Jiangsu 210023, China

2. International Institute for Earth System Science, Nanjing University, Nanjing, 210023, China

3. State Key Laboratory of Remote Sensing Science Jointly Sponsored by Beijing Normal University and Aerospace Information Research Institute, Chinese Academy of Sciences, Beijing, 100854, China

4. Jiangsu Center for Collaborative Innovation in Geographical Information Resource Development and Application, Nanjing, Jiangsu 210023, China

5. Jiangsu Key Laboratory of Agricultural Meteorology, School of Applied Meteorology, Nanjing University of Information Science and Technology, Nanjing, 210044, China

6. State Key Laboratory of Resources and Environmental Information System, Institute of Geographic Sciences and Natural Resources Research, Chinese Academy of Sciences, Beijing, 100101, China

Corresponding author(s): Zhou Yanlian ([zhouyl@nju.edu.cn)](mailto:zhouyl@nju.edu.cn))

Table S1. Site information for model calibration and validation.

| Site Name | Latitude(°) | Longitude(°) | Vegetation Type | Years | R^2^ | RMSE |
| --- | --- | --- | --- | --- | --- | --- |
| Model Calibration | | | | | | |
| CH-Oe2 | 47.29 | 7.73 | CRO | 2004-2008, 2009,2011-2013 | 0.50 | 3.40 |
| DE-Geb | 51.10 | 10.91 | CRO | 2006-2014 | 0.77 | 2.38 |
| DE-Kli | 50.89 | 13.52 | CRO | 2011-2014 | 0.69 | 2.55 |
| DE-RuS | 50.87 | 6.45 | CRO | 2006-2007,2009-2010,2012-2013 | 0.48 | 4.29 |
| US-ARM | 36.61 | -97.49 | CRO | 2011-2013 | 0.58 | 1.65 |
| US-Lin | 36.36 | -119.84 | CRO | 2010 | 0.37 | 1.02 |
| DE-Hai | 51.08 | 10.45 | DBF | 2000-2012 | 0.88 | 1.76 |
| DK-Sor | 55.49 | 11.64 | DBF | 2000-2014 | 0.94 | 1.53 |
| FR-Fon | 48.48 | 2.78 | DBF | 2006-2014 | 0.86 | 1.75 |
| IT-Isp | 45.81 | 8.63 | DBF | 2013-2014 | 0.91 | 1.62 |
| IT-PT1 | 45.20 | 9.06 | DBF | 2002-2003 | 0.90 | 1.63 |
| IT-Ro2 | 42.39 | 11.92 | DBF | 2002-2008,2010 | 0.81 | 1.96 |
| JP-MBF | 44.39 | 142.32 | DBF | 2004-2005 | 0.79 | 1.71 |
| US-Ha1 | 42.54 | -72.17 | DBF | 2000-2012 | 0.86 | 1.95 |
| US-UMB | 45.56 | -84.71 | DBF | 2000-2014 | 0.90 | 1.47 |
| US-WCr | 45.81 | -90.08 | DBF | 2000-2006,  2011-2014 | 0.89 | 1.60 |
| US-Wi3 | 46.63 | -91.10 | DBF | 2004 | 0.93 | 1.12 |
| AU-Tum | -35.66 | 148.15 | EBF | 2012-2014 | 0.52 | 3.61 |
| BR-Sa1 | -2.86 | -54.96 | EBF | 2002,2005 | 0.32 | 2.24 |
| FR-Pue | 43.74 | 3.60 | EBF | 2001-2005,2007-2009,2011-2013 | 0.43 | 1.93 |
| GF-Guy | 5.28 | -52.92 | EBF | 2007, 2009 | 0.30 | 2.36 |
| IT-Cp2 | 41.70 | 12.36 | EBF | 2012-2013 | 0.32 | 3.37 |
| CA-Man | 55.88 | -98.48 | ENF | 2000-2004,  2006-2008 | 0.66 | 1.38 |
| CA-NS1 | 55.88 | -98.48 | ENF | 2003-2005 | 0.83 | 1.05 |
| CA-NS2 | 55.91 | -98.52 | ENF | 2002-2004 | 0.88 | 0.82 |
| CA-Qfo | 49.69 | -74.34 | ENF | 2004-2007,2010 | 0.72 | 1.25 |
| CA-SF1 | 54.49 | -105.82 | ENF | 2004-2006 | 0.73 | 2.07 |
| CA-TP2 | 42.77 | -80.46 | ENF | 2003-2007 | 0.67 | 3.60 |
| CA-TP3 | 42.71 | -80.35 | ENF | 2003-2014 | 0.77 | 1.99 |
| CZ-BK1 | 49.50 | 18.54 | ENF | 2004-2014 | 0.76 | 2.46 |
| DE-Lkb | 49.10 | 13.30 | ENF | 2010-2013 | 0.80 | 0.80 |
| DE-Tha | 50.96 | 13.57 | ENF | 2000-2014 | 0.90 | 1.63 |
| FI-Hyy | 61.85 | 24.29 | ENF | 2000-2014 | 0.89 | 1.29 |
| IT-La2 | 45.95 | 11.29 | ENF | 2001 | 0.77 | 2.39 |
| IT-Lav | 45.96 | 11.28 | ENF | 2003-2014 | 0.73 | 2.97 |
| IT-Ren | 46.59 | 11.43 | ENF | 2002-2003,  2005-2013 | 0.75 | 2.23 |
| IT-SR2 | 43.73 | 10.29 | ENF | 2013-2014 | 0.82 | 1.83 |
| RU-Fyo | 56.46 | 32.92 | ENF | 2000-2014 | 0.85 | 1.69 |
| US-Blo | 38.90 | -120.63 | ENF | 2000-2006 | 0.64 | 1.62 |
| US-Me2 | 44.45 | -121.56 | ENF | 2002-2014 | 0.68 | 2.16 |
| US-Me3 | 44.32 | -121.61 | ENF | 2006,2008-2009 | 0.53 | 1.12 |
| US-Me6 | 44.32 | -121.61 | ENF | 2011,2013 | 0.54 | 1.24 |
| US-Wi4 | 46.74 | -91.17 | ENF | 2003-2004 | 0.62 | 3.18 |
| CH-Oe1 | 47.29 | 7.73 | GRA | 2002-2008 | 0.50 | 3.62 |
| CN-Du2 | 42.05 | 116.28 | GRA | 2007-2007 | 0.62 | 0.78 |
| CN-HaM | 37.37 | 101.18 | GRA | 2002, 2004 | 0.92 | 0.78 |
| CZ-BK2 | 49.49 | 18.54 | GRA | 2006-2012 | 0.78 | 1.59 |
| DE-Gri | 50.95 | 13.51 | GRA | 2001-2014 | 0.73 | 2.29 |
| DE-RuR | 50.62 | 6.30 | GRA | 2012-2014 | 0.77 | 2.12 |
| IT-Tor | 45.84 | 7.58 | GRA | 2009-2014 | 0.83 | 1.44 |
| NL-Hor | 52.24 | 5.07 | GRA | 2004-2010 | 0.83 | 1.58 |
| US-AR1 | 36.43 | -99.42 | GRA | 2009-2012 | 0.47 | 1.89 |
| US-AR2 | 36.64 | -99.60 | GRA | 2009-2012 | 0.47 | 1.09 |
| US-Wkg | 31.74 | -109.94 | GRA | 2005-2008,  2010-2014 | 0.64 | 0.75 |
| BE-Bra | 51.31 | 4.52 | MF | 2002-2014 | 0.76 | 1.85 |
| BE-Vie | 50.30 | 6.00 | MF | 2000-2014 | 0.83 | 2.02 |
| CA-TP4 | 42.71 | -80.36 | MF | 2003-2014 | 0.85 | 1.54 |
| US-PFa | 45.95 | -90.27 | MF | 2000-2014 | 0.84 | 1.12 |
| US-Syv | 46.24 | -89.35 | MF | 2001-2007,  2012-2014 | 0.80 | 1.71 |
| CA-NS6 | 55.92 | -98.96 | OSH | 2002-2005 | 0.93 | 0.51 |
| CA-SF3 | 54.09 | -106.01 | OSH | 2001-2006 | 0.72 | 0.99 |
| AU-Dry | -15.26 | 132.37 | SAV | 2009-2014 | 0.63 | 1.21 |
| CG-Tch | -4.29 | 11.66 | SAV | 2007-2009 | 0.32 | 2.84 |
| CZ-wet | 49.02 | 14.77 | WET | 2007-2014 | 0.89 | 1.25 |
| DE-Akm | 53.87 | 13.68 | WET | 2010-2013 | 0.86 | 2.20 |
| DE-SfN | 47.81 | 11.33 | WET | 2013-2014 | 0.77 | 1.15 |
| US-Myb | 38.05 | -121.76 | WET | 2011-2014 | 0.50 | 2.70 |
| AU-Gin | -31.38 | 115.71 | WSA | 2013-2014 | 0.39 | 1.00 |
| Model Validation | | | | | | |
| DE-Seh | 50.87 | 6.45 | CRO | 2008-2009 | 0.66 | 3.09 |
| FR-Gri | 48.84 | 1.95 | CRO | 2006-2007,  2009-2013 | 0.61 | 2.97 |
| US-CRT | 41.63 | -83.35 | CRO | 2011-2013 | 0.68 | 2.96 |
| CA-Oas | 53.63 | -106.20 | DBF | 2000-2010 | 0.91 | 1.46 |
| IT-Col | 41.85 | 13.59 | DBF | 2000-2014 | 0.74 | 2.69 |
| IT-Ro1 | 42.41 | 11.93 | DBF | 2001-2008 | 0.74 | 2.00 |
| US-UMd | 45.56 | -84.70 | DBF | 2008-2014 | 0.91 | 1.85 |
| BR-Sa3 | -3.02 | -54.97 | EBF | 2001 | 0.36 | 1.56 |
| IT-Cpz | 41.71 | 12.38 | EBF | 2001-2008 | 0.52 | 2.51 |
| CA-NS3 | 55.91 | -98.38 | ENF | 2002-2005 | 0.88 | 0.98 |
| CA-NS5 | 55.86 | -98.49 | ENF | 2002-2005 | 0.88 | 1.40 |
| CA-SF2 | 54.25 | -105.88 | ENF | 2003-2005 | 0.83 | 2.07 |
| DE-Obe | 50.79 | 13.72 | ENF | 2008-2014 | 0.90 | 1.56 |
| NL-Loo | 52.17 | 5.74 | ENF | 2000-2014 | 0.86 | 1.65 |
| US-GLE | 41.37 | -106.24 | ENF | 2006-2014 | 0.79 | 2.40 |
| US-NR1 | 40.03 | -105.55 | ENF | 2006-2013 | 0.85 | 2.46 |
| CH-Fru | 47.12 | 8.54 | GRA | 2006-2014 | 0.70 | 3.04 |
| IT-MBo | 46.01 | 11.05 | GRA | 2003-2013 | 0.79 | 1.99 |
| RU-Ha1 | 54.73 | 90.00 | GRA | 2003-2004 | 0.93 | 0.72 |
| CA-Gro | 48.22 | -82.16 | MF | 2004-2013 | 0.84 | 1.59 |
| CA-Obs | 53.99 | -105.12 | MF | 2000-2010 | 0.86 | 1.37 |
| CA-NS7 | 56.64 | -99.95 | OSH | 2003-2005 | 0.88 | 0.86 |
| ZA-Kru | -25.02 | 31.50 | SAV | 2001-2004,  2009-2012 | 0.67 | 2.35 |
| DE-Spw | 51.89 | 14.03 | WET | 2011-2014 | 0.93 | 1.56 |
| AU-Gin | -31.38 | 115.71 | WSA | 2012 | 0.49 | 1.12 |


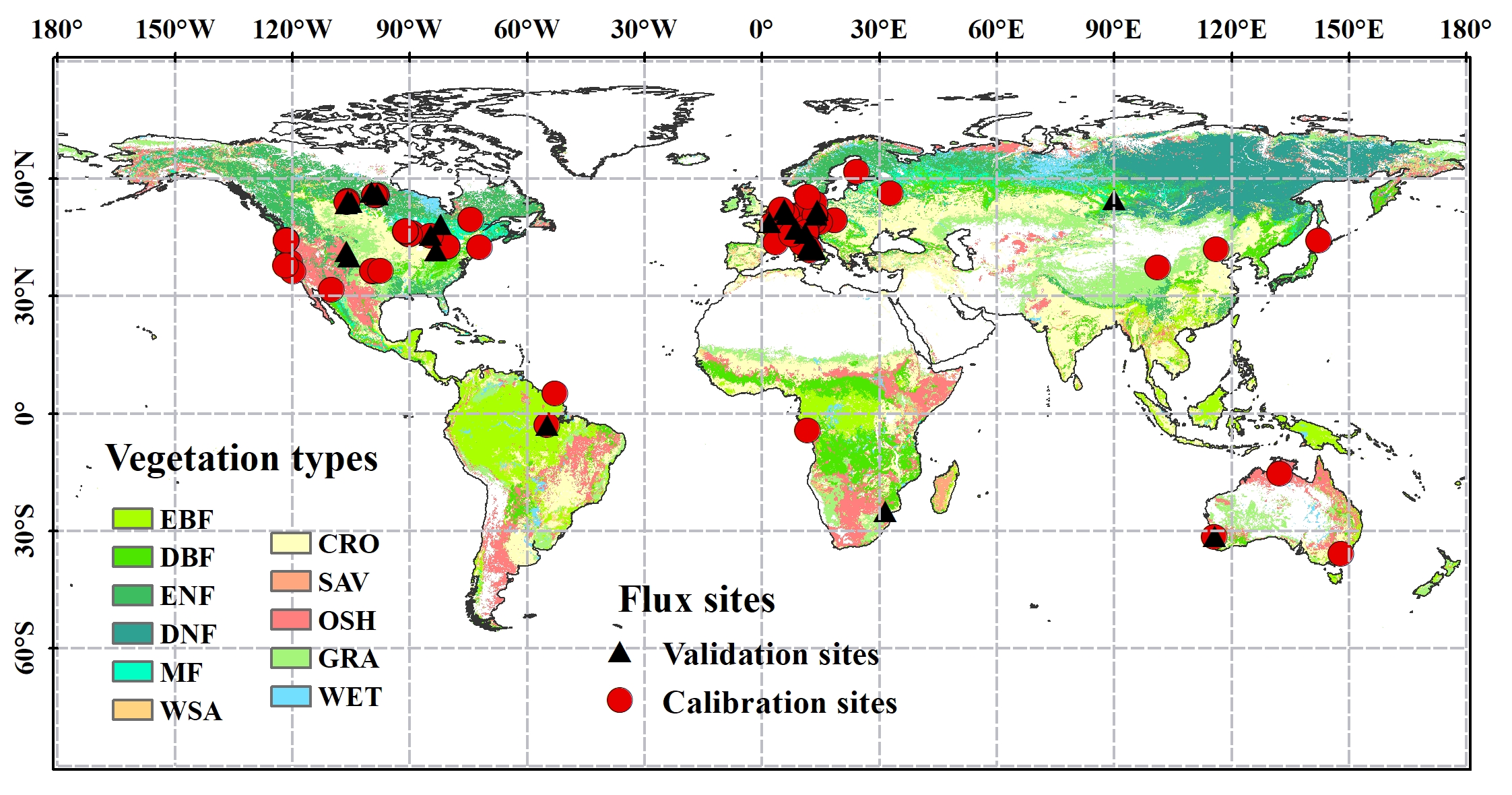


Figure S1. Distribution of FLUXNET2015 flux tower sites for calibration and validation over the globe. The background land cover map is derived from ESA-CCI land cover data in 2019.


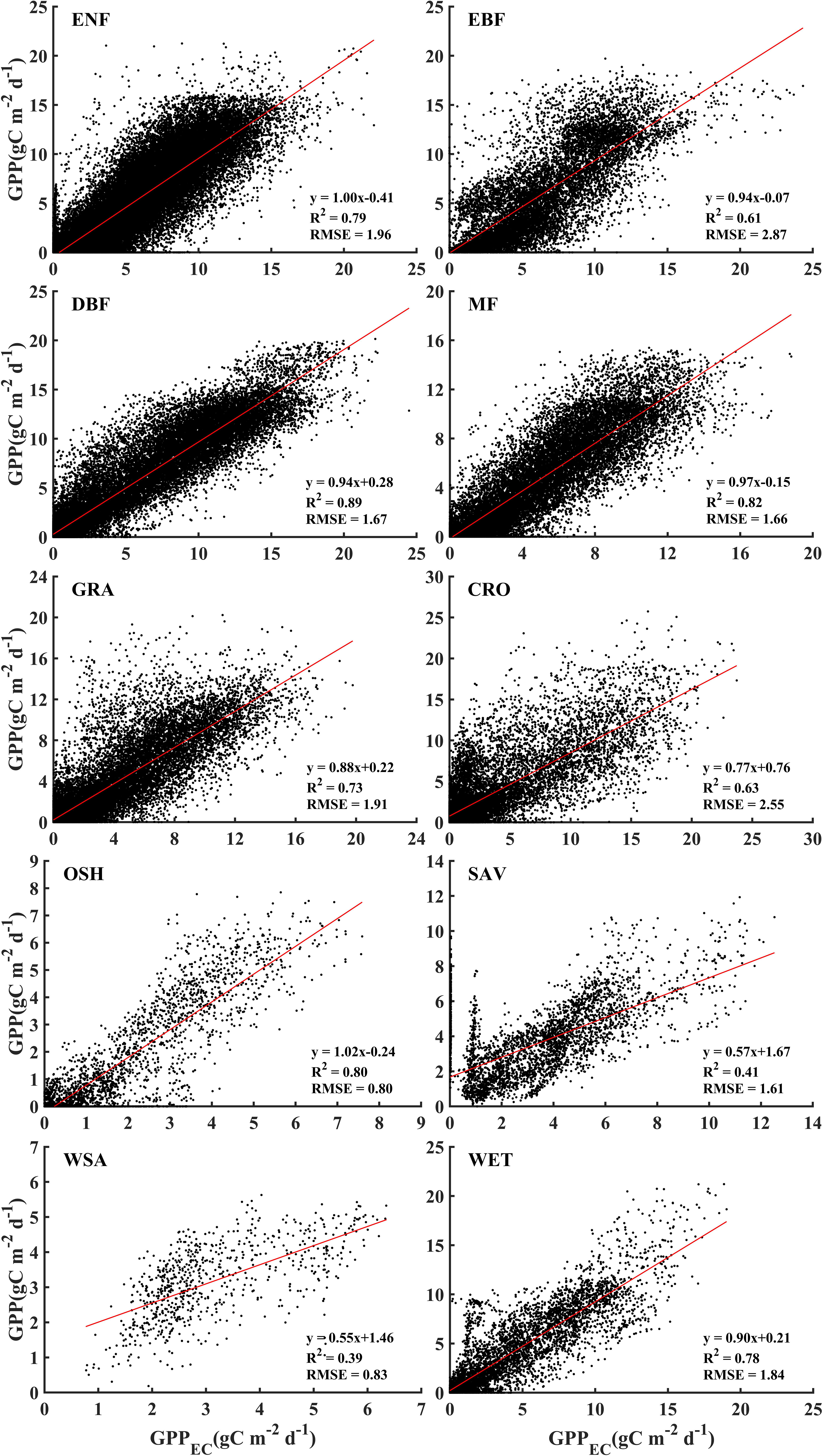


Figure S2. The result of model calibration for different vegetation types. GPP_EC_ denotes flux tower measurements, and GPP is the estimation by the improved TL-LUE model.
